# A machine learning-based framework for predicting type 2 diabetes mellitus using hematological indices

**DOI:** 10.1016/j.ahjo.2026.100854

**Published:** 2026-08-03

**Authors:** Niloufar Kamkar, Saleh Behzadi, Vahid Mahdavizadeh, Masoome Maali Tafti, Amin Mansoori, Mohadeseh Poudineh, Habibollah Esmaily, Majid Ghayour-Mobarhan

**Affiliations:** a Department of Applied Mathematics, Faculty of Mathematical Sciences, Ferdowsi University of Mashhad, P. O. Box 1159, Mashhad, 91775, Iran; b Student Research Committee, Rafsanjan University of Medical Sciences, Rafsanjan, Iran; c Department of Cardiovascular, Mashhad University of Medical Sciences, Mashhad, Iran; d Mathematical Biology Research Laboratory (MBRL), Faculty of Mathematical Sciences, Ferdowsi University of Mashhad, Mashhad, Iran.; e School of Medicine, Zanjan University of Medical Sciences, Zanjan, Iran; f Department of Biostatistics, School of Health, Mashhad University of Medical Sciences, Mashhad, Iran; g Social Determinants of Health Research Center, Mashhad University of Medical Sciences, Mashhad, Iran; h Metabolic Syndrome Research Center, Mashhad University of Medical Sciences, Mashhad, Iran

**Keywords:** Type 2 diabetes mellitus, Hematologic factors, Glomerular filtration rate (GFR), Machine learning

## Abstract

**Objective:**

This study aimed to develop a machine learning (ML) framework to predict incident type 2 diabetes mellitus (T2DM) using routinely available hematological and renal biomarkers, and to assess their added predictive value over conventional clinical risk factors.

**Methods:**

We analyzed data from 6093 diabetes-free participants from the prospective Mashhad Stroke and Heart Atherosclerotic Disorder (MASHAD) cohort. Predictors included white blood cell count (WBC), red blood cell count (RBC), red cell distribution width (RDW), and other hematological/renal factors. We employed logistic regression and multiple ML models (Random Forest, XGBoost, LightGBM), optimized via grid search and cross-validation.

**Results:**

Multivariate logistic regression identified RBC, WBC, and RDW as independent predictors of T2DM. The Random Forest model achieved the highest performance with a ROC-AUC of 0.73, an accuracy of 0.67, and correctly identified 223 of 347 incident T2DM cases Using a probability threshold of 0.20, the Random Forest model achieved a sensitivity of 0.618, a specificity of 0.707, a positive predictive value (PPV) of 0.327, and a negative predictive value (NPV) of 0.889 on the independent test set Feature importance analysis identified metabolic syndrome, BMI, uric acid, and age as the strongest contributors, while WBC, RBC, and NLR were the most influential hematological predictors.

**Conclusion:**

Routine hematological indices, including RBC, WBC, and RDW, were independently associated with incident T2DM, while metabolic syndrome, BMI, uric acid, and age contributed most strongly to overall model prediction. ML provides a complementary approach for early risk stratification, although further validation is required before clinical implementation.

## 1 Introduction

Diabetes mellitus (DM) is a chronic metabolic disorder characterized by sustained hyperglycemia resulting from impaired insulin secretion, insulin resistance, or both [1]. Among its various forms, type 2 diabetes mellitus (T2DM) is the most prevalent, accounting for the majority of diabetes cases globally [2]. Over the past few decades, the incidence of T2DM has risen substantially, primarily due to urbanization, sedentary lifestyles, and unhealthy dietary patterns [3]. As of 2017, an estimated 462 million individuals—comprising 6.28% of the global population—were diagnosed with T2DM, with the global prevalence projected to rise to 7079 per 100,000 by 2030 [4].

T2DM is associated with a wide range of complications, including cardiovascular disease, nephropathy, neuropathy, and retinopathy, all of which contribute to significant morbidity, mortality, and economic burden on healthcare systems [5], [6]. The risk factors for T2DM can be broadly categorized into non-modifiable factors, such as genetic predisposition and aging, and modifiable factors, including obesity, physical inactivity, and suboptimal dietary habits [7]. Despite notable advancements in diabetes research, the rising global prevalence of T2DM underscores the pressing need for more effective risk stratification, early diagnosis, and prevention strategies [8].

Recent research has indicated that hematological and biochemical markers may reflect early metabolic disturbances that precede clinical onset. In particular, changes in white blood cell (WBC) count, red blood cell (RBC) count, mean corpuscular volume (MCV), red cell distribution width (RDW), platelet count, platelet distribution width (PDW), and the neutrophil-to-lymphocyte ratio (NLR) have been linked to systemic inflammation and oxidative stress, both of which are implicated in the pathophysiology of diabetes [9], [10], [11], [12], [13], [14]. Furthermore, glomerular filtration rate (GFR) and serum uric acid levels, as markers of renal function and metabolic health, have shown associations with insulin resistance and diabetes risk [9], [15], [16]. While previous research has established associations between hematological markers and T2DM, their predictive utility remains underexplored, particularly in large-scale cohort studies. Recent studies also link platelet indices, RDW, and other CBC parameters with glycemic deterioration and vitamin D status, suggesting a broader hematologic-metabolic interplay [17], [18], [19].

Traditional diagnostic tools—including fasting plasma glucose, glycated hemoglobin (HbA1c), and oral glucose tolerance tests—are useful but may not detect early, subclinical metabolic changes. In this context, machine learning (ML) offers a promising alternative by identifying complex, nonlinear patterns in large datasets. Leveraging ML algorithms, this study aims to evaluate the predictive potential of selected hematological and renal biomarkers—WBC, RBC, MCV, RDW, PDW, platelet count, NLR, GFR, and serum uric acid—for T2DM within a cohort study framework.

## 2 Methods and materials

### 2.1 Study design

**Fig. 1:**
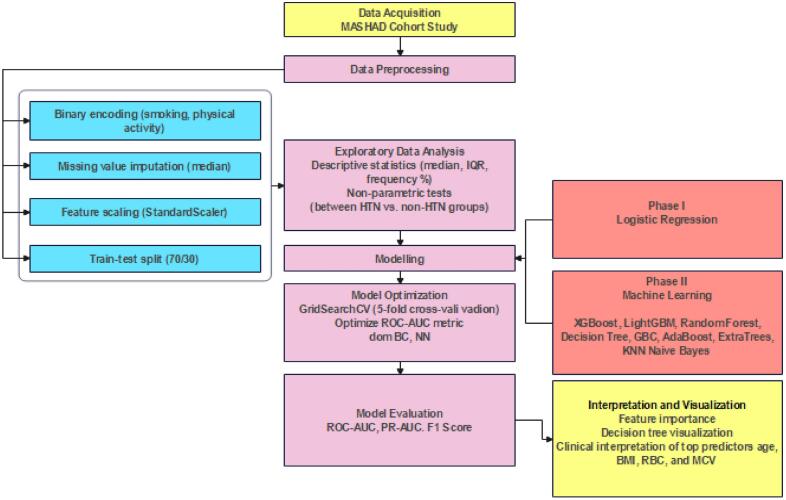
Schematic representation of the overall study workflow.

The present study was conducted on a database derived from the Mashhad Stroke and Heart Atherosclerotic Disorder (MASHAD) study, a cohort study conducted in Mashhad, Iran [20]. In short, the cohort began in 2010 and has continued to 2020 and included data on demographic, anthropometric, lifestyle, and laboratory measurements. Using stratified cluster random sampling, the initial number of participants in the MASHAD study was 11,247. However, after the removal of cases due to non-response, and a prevalent history of cardiovascular diseases, the final number of patients was 9704. Finally, after removing those with a previous history of diabetes mellitus at baseline, the final number of included subjects reached 6093. From which 1156 participants diagnosed as T2DM. The data collection in the MASHAD study was conducted via interview. Gender, age, and race were based on self-reporting. Height and weight were assessed during the physical assessment appointments utilizing conventional measurement devices and techniques. Additionally, systolic and diastolic blood pressure readings were the averages of the second and third consecutive tests, while applying the criteria for standard measurements. The medical, pharmaceutical, and social histories of the participants were acquired during interview sessions conducted by two trained healthcare professionals and a nurse. Additionally, the biochemical analyses of patients were conducted using blood and mid-stream urine samples. Current smoking status was described as smoking at least once daily, and non-smoker status was defined as no history of smoking or a history of smoking but with no longer engagement. The outcome of interest in this study was the occurrence of type II diabetes mellitus on follow-up, defined by an FBG ≥ 126 mg/dL or being treated with existing oral hypoglycemic agents or insulin. Participants with diabetes at baseline were excluded to ensure that only incident diabetes cases were evaluated. Participants were followed within the MASHAD cohort between baseline assessment and subsequent follow-up visits conducted during the study period (2010−2020). Individuals who did not meet the diabetes definition during follow-up were considered non-cases at the end of follow-up. Because the present analysis focused on incident diabetes status rather than time-to-event analysis, the exact timing of diabetes onset and censoring times were not incorporated into the prediction models. Fig. 1 illustrates the schematic overview of the study workflow.

### 2.2 Data preprocessing

Data preprocessing involves transforming raw data into a format that is suitable for analysis and modeling. Thus, the predictions of the model become more reliable. In this study, the data preprocessing was implemented in a structural pipeline with four consecutive steps to resolve inconsistencies, outliers and missing values and make the model more reliable.

Initially, binary encoding is implemented to encode categorical data — defined as variables that belong to distinct categories such as labels, names or types — into numerical form that models can interpret. Given that most ML algorithms require numerical inputs, encoding categorical data into numerical data in an appropriate manner gives rise to accurate and meaningful interpretation, bias reduction and enhanced predictive accuracy. Consequently, we transformed categorical features into binary classifications for better interpretability. Therefore, smoking was classified as non-smoking (with no history or a past-smoking history) and current smoking, job status into employed and non-employed, and physical activity into high and non-high classes.

Subsequently, the SimpleImputer class was employed to impute missing values during the preprocessing process. The “median” was chosen as an imputation strategy for the continuous features as a result of the non-normal distribution of the data. Using the median provides a more reliable measure of central tendency for skewed distributions. This approach helps prevent bias in the imputation process and preserves the original statistical properties of the features as much as possible.

After handling categorical and missing data, the StandardScaler was implemented on the training set to standardize the spectrum of features. It is generally used to transform the data distribution by subtracting the mean and dividing by the standard deviation. This process centers the data around zero and scales it to have a standard deviation of 1 [21].

Finally, the dataset was divided into a 70% train and a 30% test sets using a constant method to guarantee that the models were evaluated on the same individuals in the test set. The train set is a subset of the given data that is utilized to fit the model and on which the model is trained, while the test set is a separate portion of the overall data, used to evaluate the performance of the final model on unseen (independent) data.

### 2.3 Exploratory data analysis (EDA)

Following the preprocessing step (with the aim of handling missing values, encoding categorical features, and standardizing numerical variables), EDA was conducted to examine the underlying structure and characteristics of the cleaned dataset before applying ML algorithms or statistical modeling.

Continuous data are summarized based on the shape of their distribution. For instance, if the data are normally distributed, the mean is used to summarize the data, as it accurately represents the center of normally distributed data and adequately reflects the entire dataset. In contrast, for non-normally distributed data, the median is used because it is more reliable in representing the central value of the data. Moreover, the interquartile range (IQR), a measure of statistical dispersion representing the spread of the middle 50% of the data [22], is applied identify the data distribution pattern. Because of the non-normal distribution of the data in this study, continuous variables were denoted as the median with the interquartile range (IQR). The study sample was classified according to whether participants developed incident T2DM during follow-up or remained free of diabetes throughout the follow-up period then, the continuous and categorical features were compared between the groups within each classification using non-parametric tests.

### 2.4 Statistical analysis

The analyses in this study were conducted in two distinct phases. The first phase focused on logistic regression as a fundamental statistical method and in the second phase, more complex ML algorithms were used to identify features with a considerable contribution to the prediction of developing diabetes in Table 1. Table 2 summarizes the final hyperparameter settings and provides a brief description of each ML classifier. Random Forest was selected as the final model because it demonstrated the best overall discrimination performance on the independent test set.

**Table 1:** ML models and algorithms utilized in the proposed framework for Predicting T2DM.

Algorithms	Description
Stratified cluster sampling	Stratified cluster sampling is a statistical sampling technique integrating stratified and cluster sampling. In this approach, the population is first divided into different strata based on specific characteristics in order to maintain diversity. Then some clusters are selected randomly from each stratum, which simplifies data collection [54].
Logistic regression	Logistic regression is a statistical procedure used to quantify the relationship between one dependent and one or more independent variables (multivariate logistic regression) [55].
XGBoost	XGBoost is an optimized gradient boosting algorithm that implements decision trees as its base learners. Then combines them sequentially to improve model's performance. Each new tree is trained to correct the errors of the previous tree and this process is called boosting.
LightGBM	It is an ensemble learning approach based on the gradient boosting algorithm, which iteratively combines multiple weak learners, each correcting the errors of its predecessors, to build a robust and accurate predictive model.
Decision tree	Decision tree models use a tree-like structure to visualize decisions and related outcomes, making predictions more sense to understand. The tree consists of a root node, and internal nodes showing decision rules, while branches showing rule outcomes, and leaf nodes indicating the predicted class or value. They can be applied to both classification and regression tasks [56].
Random forest	Random Forests is an ensemble learning method that builds multiple decision trees and combines their outputs to improve prediction accuracy and stability. This technique uses internal metrics to monitor error, strength, and correlation, enabling adjustments when the number of features used for splitting increases [57].
Gradient boosting classifier	Gradient boosting is a prominent machine learning technique that provides predictive models as an ensemble of weak learners, typically decision trees. It sequentially converts weak learners into strong ones, with each new model trained to correct the residual errors of the previous ensemble, thereby iteratively minimizing a loss function and improving overall predictive performance [58].
K-nearest neighbors	K-Nearest Neighbors (KNN) is a supervised machine learning algorithm generally used for classification but can also be used for regression tasks. Initially, it finds the k closest data points which is called also neighbors to a given input and then makes predictions based on the average value for regression and majority class for classification.
Naive Bayes	Naive Bayes is a supervised machine learning algorithm based on the Bayes' theorem. This approach is based on the assumption that all features are independent of each other. In the sense that each feature does not affect any other feature while classifying. This algorithm is easy to implement and computationally efficient and also effective in cases with a large amount of data.
ExtraTrees	The Extremely Randomized Trees Classifier (Extra Trees Classifier) is an ensemble learning technique that constructs multiple decision trees with randomly selected features and thresholds. The final prediction is obtained by aggregating the outputs of these de-correlated trees, which helps reduce variance and improve model generalization.
AdaBoost	AdaBoost (Adaptive Boosting) is an ensemble learning technique that integrates weak models (typically decision trees of low depth) sequentially to create a robust model. In each iteration, the weights on the misclassified samples are increased so that the subsequent model can focus on the challenging instances. This enhances the predictive power of the model. This technique is very popular because it is simpler and more effective in addressing classification problems.

**Table 2:** Hyperparameter settings for machine learning classifiers.

Estimator	Hyperparamters
XGBoost classifier	learning_rate: 0.01, max_depth: 3, n_estimators: 200, subsample: 0.8
LightGBM classifier	learning_rate: 0.01, max_depth: 3, n_estimators: 200, subsample: 0.8
Random forest classifier	learning_rate: N/A, max_depth: 3, min_samples_split: 10, n_estimators: 200, subsample: N/A
GBC classifier	learning_rate: 0.01, max_depth: 3, n_estimators: 200, subsample: 0.8
Decision tree classifier	learning_rate: N/A, max_depth: 3, min_samples_leaf: 4, min_samples_split: 2, subsample: N/A

N/A indicates a hyperparameter that is not defined or applicable for the given model architecture.

#### 2.4.1 Phase 1: logistic regression

Logistic regression analysis was conducted as an independent statistical analysis to evaluate the associations between selected hematological markers and incident T2DM after adjustment for potential confounding variables. The purpose of this analysis was to estimate the Odds Ratios (ORs) and identify factors independently associated with diabetes occurrence. In order to minimize the risk of multicollinearity, the selected features were assessed in terms of the variance inflation factor (VIF). A VIF threshold of 5 was used to identify potentially problematic multicollinearity. All predictors showed VIF values below this threshold; therefore, no variables were excluded because of multicollinearity. In addition, Spearman correlation analysis among the principal predictors used in the Random Forest model demonstrated only weak-to-moderate pairwise correlations, with the highest correlation found between metabolic syndrome and BMI. The results are shown in Table 3 and Fig. S1 in Supplementary.

**Table 3:** Variance inflation factor (VIF) for main covariates and confounding variables.

Feature	VIF
*Main covariates*	
WBC	1.23
RBC	1.99
MCV	1.60
Platelets	1.49
RDW	1.32
PDW	1.28
NLR	1.06

*Confounding variables*	
Sex	3.06
Job status	1.30
Smoking status	1.08
BMI	2.87
Waist circumference	2.35
History of hypertension	1.18
GFR	1.44
Physical activity	1.68
Uric acid	1.36

#### 2.4.2 Phase 2: machine learning

ML models were developed independently from the logistic regression analysis to evaluate the predictive performance of different algorithms for incident T2DM. This study employed Python 3.11, Jupyter Notebooks [23], SciPy 1.11.4, Scikit Learn module 1.2.2, Pandas 2.1.4, NumPy 1.26.4, XGBoost 2.1.3, and LightGBM 4.5.0 for its ML workflow. The results were presented graphically using Matplotlib 3.7.5 and Seaborn 0.13.2. In this investigation, we implemented a comprehensive ML pipeline to forecast the target variable based on a diverse array of features. Predictor variables were selected a priori based on clinical relevance, previous literature, and data availability within the MASHAD cohort.

In order to optimize the model parameters, GridSearchCV with 5-fold cross-validation was used for hyperparameter optimization. The models were optimized for the ROC-AUC metric to ensure that we focused on maximizing the model's capacity to differentiate between the positive and negative classes. The hyperparameters are shown in Table 2. Because the outcome was moderately imbalanced, stratified sampling was used during both train–test splitting and cross-validation to preserve class proportions. After identifying the optimal hyperparameters, the selected models were further evaluated using 10-fold cross-validation on the training dataset to obtain a more robust estimate of model performance before final evaluation on the independent test set. Finally, the test set was reserved for final evaluation on unseen data. The independent test set was not involved in model selection, hyperparameter tuning, or cross-validation and was used only once for the final performance evaluation.

To prevent data leakage, all preprocessing steps, including median imputation and feature standardization, were embedded within a scikit-learn Pipeline and performed independently within each cross-validation fold. Consequently, information from the validation folds or the independent test set was not used during model training or preprocessing.

In addition to discrimination metrics, model calibration was evaluated on the independent test set using calibration curves, the Brier score, calibration intercept, and calibration slope. These measures were used to assess the agreement between predicted probabilities and observed outcomes.

In the initial phase of model training, we assessed a diverse array of ML models, such as XGBoost, LightGBM, Logistic Regression, Decision Tree, Random Forest, Gradient Boosting Classifier, k-Nearest Neighbors, Naive Bayes, ExtraTrees, and AdaBoost. We identified the four most effective classifiers: XGBoost, LightGBM, Random Forest, and Gradient Boosting, following an evaluation of all models. Also, the Decision Tree estimator was selected for plotting a decision tree. These models exhibited the most favorable overall performance based on the evaluated performance metrics and were subsequently employed for a more thorough analysis.

### 2.5 Model interpretation

To gain insight into the most influential predictors, we visualized feature importance scores for tree-based models. In order to enhance comprehension of the model's decision-making process, feature importance plots were generated. A graphical representation of the tree was provided to improve the interpretability of the Decision Tree model.

Feature importance scores were interpreted as measures of the contribution of each variable to model predictions and were not considered indicators of independent clinical importance or causal relationships.

## 3 Results

This study compared different demographic and clinical factors between the subjects with and subjects without diabetes in follow-up. The results showed that males with diabetes were significantly fewer than non-diabetic males, with 415 (6.9%) diabetic males compared to 2069 (33.9%) non-diabetic males (*p* < 0.001). Such a trend was observed for females as well (*p* < 0.001). The median age of patients with diabetes was significantly higher than those without (49 years, IQR 11 versus 46 years, IQR 12, respectively) (p < 0.001). The median BMI for patients with diabetes was 29.61 (IQR 5.71) compared to non-diabetic subjects (26.97, IQR 5.90), showing a significant difference (*p* < 0.001). A similar pattern was observed in the job status category, with more non-diabetic subjects being employed (2036, 33.4%) compared to those with diabetes (382, 6.2%) (p < 0.001) (Table 4).

**Table 4:** The baseline characteristic of subjects with and without T2DM.

		Diabetic	Non-diabetic	*p*-Value
Sex	Male	415 (6.9)	2069 (33.9)	<0.001
	Female	741 (12.1)	2868 (47.1)	
Age, *median* (*IQR*)		49 (11)	46 (12)	<0.001
BMI, *median* (*IQR*)		29.61 (5.71)	26.97 (5.90)	<0.001
Job-status	Employed	382 (6.2)	2036 (33.4)	<0.001
	Unemployed	638 (10.5)	2491 (40.9)	
	Retired	136 (2.3)	410 (6.7)	
Smoking status	Non-smoker	805 (13.2)	3499 (57.5)	0.25
	Ex-smoker	116 (1.9)	420 (6.9)	
	Current smoker	235 (3.8)	1018 (16.7)	
History of hypertension		448 (7.4)	1204 (16.8)	<0.001
Metabolic syndrome		535 (8.8)	1013 (16.6)	<0.001
GFR, *median* (*IQR*)		123.82(36.93)	119.36 (34.24)	<0.001
WBC, *median* (*IQR*)		6 (1.8)	5.7 (1.8)	<0.001
RBC, *median* (*IQR*)		4.85, IQR: 0.59	4.79, IQR: 0.63	<0.001
MCV, *median* (*IQR*)		85.70, IQR: 4.90	85.90, IQR: 5.00	0.007
Platelets, *median* (*IQR*)		231 (75)	223 (70)	<0.001
RDW, *median* (*IQR*)		41.60 (3.80)	41.70 (3.60)	0.02
PDW, *median* (*IQR*)		12.20 (2.40)	12.40 (2.30)	0.01
Uric acid, *median* (*IQR*)		4.90 (1.90)	4.50 (1.70)	<0.001
NLR, *median* (*IQR*)		1.47 (0.69)	1.50 (0.69)	0.02

Smoking status was not significantly different between the groups (*p* = 0.25). However, the two groups showed significant difference in terms of a history of hypertension and metabolic syndrome (p < 0.001 for both). Furthermore, the median GFR for diabetics was 123.82 (IQR 36.93), higher than that of non-diabetics (119.36, IQR 34.24) (p < 0.001). Comparing hematological indices between the two groups also showed significant differences in WBC, RBC, platelets, and uric acid, with diabetics generally exhibiting higher values in most of these parameters (Table 4).

Performing a univariate logistic regression showed that all of the included hematological indices (i.e., WBC, RBC, MCV, Platelets, RDW, PDW, and NLR) were significantly associated with the outcome. However, after performing a multivariate logistic regression and adjusting for confounding variables (sex, job status, smoking status, BMI, waist circumference, history of hypertension, GFR, physical activity, and uric acid), only RBC, WBC, and RDW remained as significantly associated with the outcome. RBC had an OR of 1.428 (95% CI: 1.155–1.767), WBC an OR of 1.106 (95% CI: 1.046–1.169), and RDW, an OR of 0.957 (95% CI: 0.930–0.985) (Fig. S1 in Supplementary).

Training the models and then ranking them based on their performance resulted in the selection of four models. The Random Forest Classifier was ranked 1st, by yielding a ROC-AUC of 0.73 (95% CI: 0.70–0.76), PR-AUC of 0.36 (Fig. S2 in Supplementary), an accuracy of 0.67, an F1 score of 0.43, and a PR-AUC of 0.36. Furthermore, the Random Forest model successfully captured 223 positives out of a total of 347 (Fig. S3 in Supplementary). The detailed report of the performance metrics for all the models is shown in Table 5. Accordingly, it is notable that all of the models showed consistent performance between the training and the test set, showing very minimal overfitting.

**Table 5:** The performance results of the best-trained models.

Estimator	Train			Train			
	CV ROC-AUC (95%CI)	Accuracy	F1	ROC-AUC (95% CI)	Accuracy	F1	PR-AUC
Random forest classifier	0.70	0.67	0.42	0.73 (0.70–0.76)	0.67	0.43	0.36
LightGBM classifier	0.69	0.69	0.40	0.72 (0.69–0.75)	0.71	0.42	0.35
GBC classifier	0.70	0.65	0.41	0.72 (0.69–0.75)	0.69	0.42	0.36
XGBoost classifier				0.72 (0.69–0.75)			
Decision tree classifier				0.70 (0.67–0.73)			

For comparison, a baseline clinical logistic regression model including conventional clinical risk factors was also evaluated. The baseline model achieved a ROC-AUC of 0.70 (95% CI: 0.68–0.73), whereas the Random Forest model achieved a ROC-AUC of 0.73 (95% CI: 0.70–0.76). This modest improvement suggests that the inclusion of hematological markers provides additional predictive information beyond conventional clinical risk factors.

Using a probability threshold of 0.20, the Random Forest model achieved a sensitivity of 0.618, a specificity of 0.707, a positive predictive value (PPV) of 0.327, and a negative predictive value (NPV) of 0.889 on the independent test set.

**Fig. 2:**
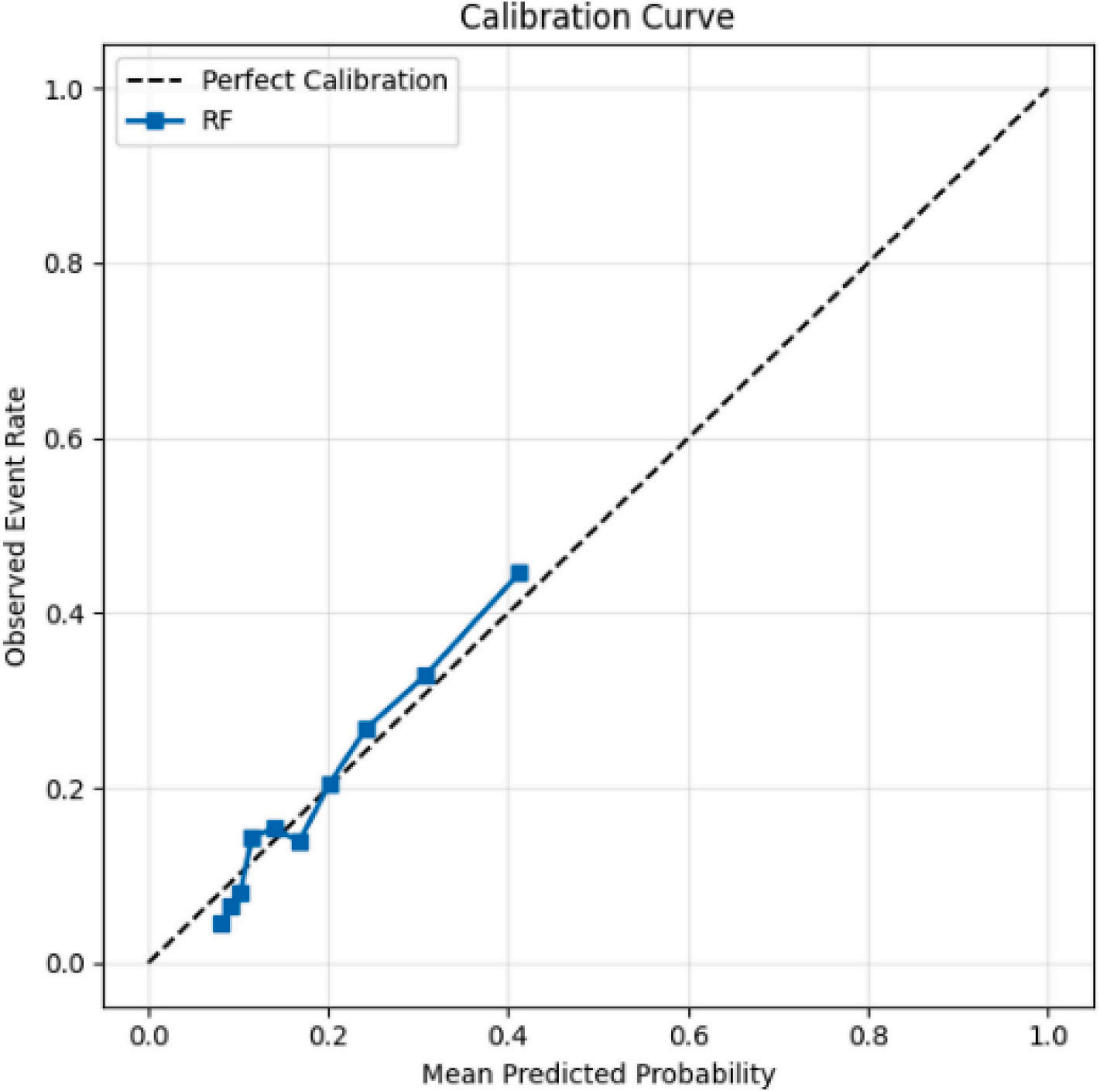
Calibration curve of random forest model for type 2 diabetes mellitus.

Calibration analysis demonstrated acceptable agreement between predicted and observed probabilities (Fig. 2). The model achieved a Brier score of 0.138, with a calibration intercept of 0.223 and a calibration slope of 1.16, indicating overall acceptable calibration despite some deviation from ideal calibration.

**Fig. 3:**
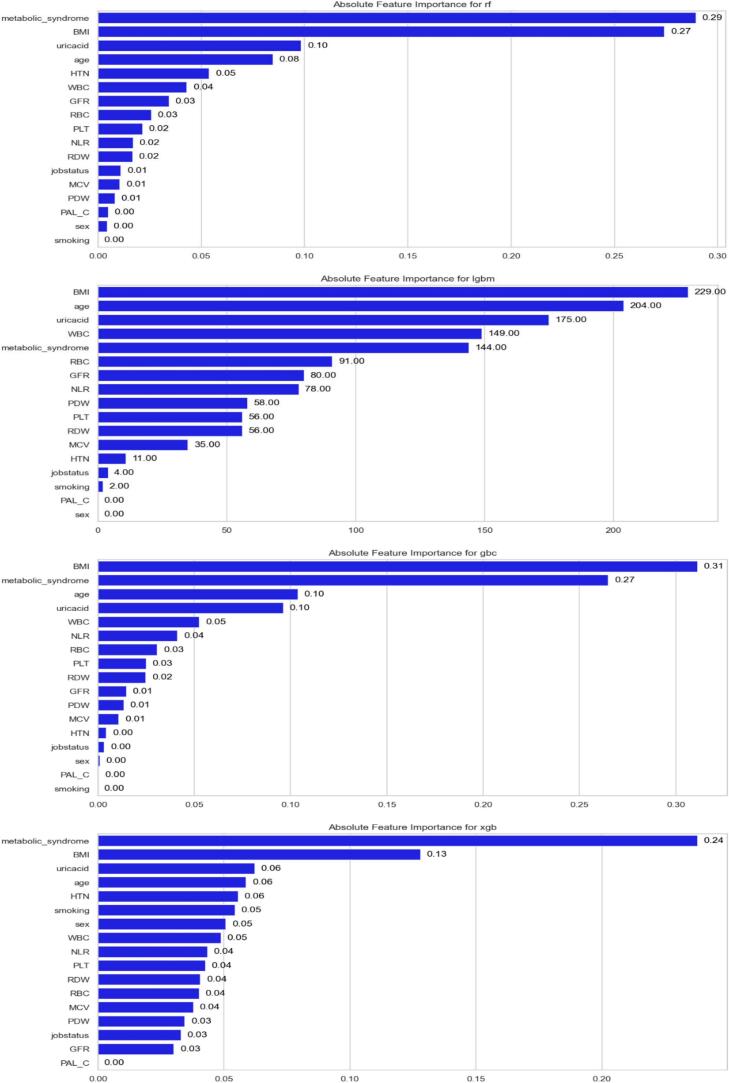
Absolute feature importance for the Random Forest (rf), LightGBM (lgbm), Gradient Boosting Classifier (gbc), and XGBoost (xgb) model.

Fig. 3 shows the feature importance plots for the selected models. Accordingly, the Random Forest identified metabolic syndrome, BMI, uric acid, and age as the top four contributing factors. The other estimators also showed relatively similar importance for these features. Among the hematological indices, WBC was placed sixth in Random Forest, fifth in GBC, and fourth in LightGBM. RBC and NLR were the next leading hematological indices in terms of contribution to the model. Also, for a clinically-friendly presentation of the models, a decision tree was plotted in this study, shown in Fig. S4 in Supplementary. According to this plot, metabolic syndrome was the first important factor in better-classifying patients. In one of the branches, the importance of NLR is shown: 62% of patients were diabetic if metabolic syndrome was present, BMI ≤ 28.07, and NLR > 0.83.

## 4 Discussion

This study identified significant demographic and clinical differences between diabetic and non-diabetic subjects. Diabetic patients were fewer in number, older, and had a higher BMI compared to non-diabetic individuals (*p* < 0.001). Hypertension and metabolic syndrome were significantly more prevalent among diabetics (p < 0.001). Although smoking status did not differ significantly, diabetics exhibited higher GFR and hematological indices, including WBC, RBC, and platelets. Multivariate logistic regression confirmed that RBC, WBC, and RDW remained independently associated with diabetes. ML models were evaluated, with the Random Forest Classifier demonstrating the best predictive performance (ROC-AUC: 0.73). Feature importance analysis highlighted metabolic syndrome, BMI, uric acid, and age as key contributors to diabetes classification, with WBC, RBC, and NLR also playing notable roles. A decision tree visualization further emphasized metabolic syndrome and NLR as critical classifiers in diabetes prediction. Because metabolic syndrome, obesity, age, and inflammatory markers are well-established cardiovascular risk factors, these findings may also contribute to cardiometabolic risk stratification in individuals at increased risk of T2DM.

Compared to our 2023 study [24], the current findings show both consistency extension of the MASHAD cohort findings. The previous publication reported the Phase I (baseline) analysis of the cohort, whereas the present study reports the Phase II follow-up, evaluating incident T2DM among participants who were free of diabetes at baseline. Age and WBC were significant in both studies, while the present analysis also identified RBC and RDW as independent predictors. Unlike the earlier focus on TyG index associations, this study applied ML, with Random Forest highlighting metabolic syndrome, BMI, uric acid, age, WBC, and RBC as key features. PDW and MCHC, previously significant, did not retain predictive value. Thus, this study provides longitudinal evidence from a later phase of the cohort and identifies hematological indices associated with incident diabetes over follow-up.

Our study demonstrated that elevated levels of RBC, WBC, and RDW were associated with the presence of T2DM after adjusting for confounding factors. These findings represent statistical associations and should not be interpreted as evidence of causal mechanisms. Previous prospective and experimental studies have suggested that chronic hyperglycemia and systemic inflammation may influence hematological indices, whereas a previous observational study involving 120 patients with T2DM observed changes in RBC and RDW levels [25]. Similarly, a recent study by Shahmoradi, based on data from the Iranian Bandare-Kong Non-Communicable Diseases (BKNCD) cohort, reported a positive association between WBC count and diabetes, aligning with our findings. However, their inverse RDW association contrasts with ours, possibly due to population or glycemic control differences. Jaman et al. also connected high RDW with poor glycemic control [26]. Jaman et al. also connected high RDW with poor glycemic control [27]. A systematic review of over 14,000 individuals found elevated WBC and altered leukocyte profiles in diabetics, though RBC changes varied by study [28].

The Random Forest Classifier achieved the best predictive performance among the evaluated models, consistent with several studies that have highlighted its strength in diabetes prediction tasks. For instance, a study comparing five ML algorithms for diabetes prediction found higher AUC with XGBoost, but Random Forest stood out for its balanced performance and greater interpretability [29]. It has previously achieved high AUCs [30], outperformed logistic regression in modeling HbA1c trends [31], and effectively identified key predictors like BMI [32], [33], [34], [35]. Metabolic syndrome, BMI, uric acid levels, and age showed the highest contribution to the predictions generated by the Random Forest model. This aligns with previous studies linking uric acid levels to T2DM risk [36], [37]. Although Li et al. suggested a potential causal relationship between hyperuricemia and insulin resistance [38], our findings only demonstrate that uric acid contributed to prediction and do not provide evidence regarding causality. In contrast, mendelian randomization studies have found no evidence for a causal link [39], [40]. The association between metabolic syndrome, elevated BMI, and T2DM risk is well-documented [41], [42], [43]. Importantly, these variables are also established determinants of cardiovascular risk, supporting the potential relevance of our prediction model for broader cardiometabolic risk assessment.

Compared with the baseline clinical logistic regression model, the Random Forest model demonstrated a modest improvement in discrimination, suggesting that hematological markers may provide incremental predictive information beyond conventional clinical risk factors. Whether this incremental predictive value translates into improved cardiovascular risk prediction requires validation in studies with cardiovascular outcomes.

From a clinical perspective, the probability threshold represents a trade-off between sensitivity and specificity. Lower thresholds may identify more individuals at risk but increase false-positive predictions, whereas higher thresholds may improve specificity at the expense of missing true cases.

Age was a strong predictor of T2DM in our model, aligning with findings from several studies, including a case-control study in Trinidad [44]. Other researches have also reported similar associations [24], [45], [46]. Though some research suggests its influence may vary by population or study design [47], [48].

Our findings support the link between hematological markers—especially WBC, RBC, and NLR—and T2DM in multivariate logistic regression analysis, echoing studies connecting them to insulin resistance and beta-cell dysfunction [49], [50]. However, our predictive model identifies variables that improve classification performance rather than establishing the biological pathways responsible for diabetes development. Though Ouda et al. found elevated inflammatory markers, their results weren't significant [51]. Consistency with Shahmoradi et al.'s large-scale data [26] highlights the potential utility of these markers as predictors within this cohort, while also supporting the recognized inflammatory component of diabetes. Evidence on NLR remains mixed, with some studies confirming an association [52], [53], and others finding none [52].

This study has several notable strengths. By combining multivariate logistic regression with ML techniques, we robustly identified independent hematological predictors of T2DM while adjusting for key confounders. The dual approach enhanced both the interpretability and predictive accuracy of our findings. Additionally, grounding our results in previous literature, including our prior analysis, provided a more comprehensive understanding of hematological dynamics in diabetes. The use of readily available hematological and metabolic variables may also facilitate future development of practical cardiometabolic risk prediction tools, although this application requires external validation.

Although some predictors were biologically related, both the VIF assessment and the correlation analysis indicated limited multicollinearity. Therefore, correlated predictors were unlikely to substantially affect model stability, although moderate correlations may slightly influence the interpretation of individual feature importance.

However, certain limitations must be acknowledged. The prospective cohort design allows temporal assessment of incident T2DM but remains observational; therefore, causal relationships cannot be inferred. Additionally, although the MASHAD cohort is a prospective cohort, the present analysis was based on available follow-up information and did not include repeated longitudinal measurements of all predictors over time, which may limit the assessment of changes in risk factors and their dynamic relationship with T2DM development. Our findings are based on data from a single perspective cohort, limiting their generalizability beyond populations with similar demographic and clinical characteristics. Moreover, residual confounding due to unmeasured variables—such as undiagnosed inflammatory or hematological disorders—remains a possibility. In addition, although several metabolic and clinical variables were included, some potentially relevant predictors, such as individual blood glucose measurements and other metabolic parameters, were not available for analysis. The absence of these variables may have limited the comprehensiveness of the prediction model and should be addressed in future studies. Although the outcome distribution was moderately imbalanced, stratified train–test splitting and stratified cross-validation were applied throughout model development to preserve class proportions and support robust model evaluation. Future studies should adopt longitudinal, multi-center designs to validate our findings and further elucidate the biological mechanisms underlying these associations. Future studies should adopt longitudinal, multi-center designs to validate our findings and further elucidate the biological mechanisms underlying these associations. In addition, external validation in independent cohorts and evaluation of cardiovascular outcomes will be important to determine the broader clinical utility of the proposed prediction model. We did not include vitamin D or detailed platelet indices beyond PDW; prior work has shown these markers relate to glycemic control and vascular risk and might improve predictive models [17], [18], [19].

## 5 Conclusion

Our findings demonstrate significant differences in clinical and hematological indices between diabetic and non-diabetic individuals, reinforcing the link between metabolic dysfunction, inflammation, and T2DM development. Logistic regression analysis identified elevated RBC and WBC, along with reduced RDW, as independent predictors of T2DM. ML models, particularly the Random Forest Classifier, further validated these associations, achieving a ROC-AUC of 0.73. Among all evaluated predictors, metabolic syndrome, BMI, uric acid, and age were the strongest contributors to model performance, whereas hematological markers, including WBC, RBC, and NLR, provided additional predictive information beyond conventional clinical risk factors. These findings suggest that routinely available hematological indices may complement established metabolic and clinical predictors rather than replace them in early T2DM risk assessment. The integration of ML provides a complementary framework for risk stratification and may support future cardiometabolic risk prediction after external validation. Future research should validate these findings in independent and diverse populations and determine whether incorporating hematological indices meaningfully improves clinical prediction models for diabetes prevention.

## CRediT authorship contribution statement

**Niloufar Kamkar:** Writing – original draft. **Saleh Behzadi:** Writing – original draft. **Vahid Mahdavizadeh:** Software, Formal analysis, Conceptualization. **Masoome Maali Tafti:** Writing – original draft. **Amin Mansoori:** Writing – review & editing. **Mohadeseh Poudineh:** Writing – original draft. **Habibollah Esmaily:** Formal analysis. **Majid Ghayour-Mobarhan:** Writing – review & editing.

## Consent for publication

All the participants consented to take part in the study by signing written informed consent. Also, it is not applicable to the Consent of Image Publication for this manuscript. The figures were designed only in this manuscript for presenting the results of the current paper.

## Ethics approval and consent to participate

All participants provided written informed consent to take part in the study. The original study protocol was reviewed and approved by the Ethics Committee of Mashhad University of Medical Sciences (approval number: IR.MUMS.REC.1386.250) on July 2007. The current secondary analysis was conducted under the scope of this original approval and does not require additional ethical review. All methods were carried out in accordance with relevant guidelines and regulations.

## Funding

This research received no specific grant from any funding agency in the public, commercial, or not-for-profit sectors.

## Declaration of competing interest

The authors declare that they have no known competing financial interests or personal relationships that could have appeared to influence the work reported in this paper.

## Data Availability

The datasets used and/or analyzed during the current study available from the corresponding author on reasonable request.
