# Supplementary material for: A machine learning-based framework for predicting type 2 diabetes mellitus using hematological indices
Source: Am Heart J Plus. 2026 Aug 3;69:100854. doi: 10.1016/j.ahjo.2026.100854 (PMC13475539; doi:10.1016/j.ahjo.2026.100854)
Supplement: Supplementary file 1 [file mmc1.docx]

Figure S1. The Radar Plot for Univariate (Blue) and Multivariate (Red) Analysis and Odds Ratios for Features.

Figure S2. Receiver Operating Characteristic (ROC) Curve and Precision-Recall Curve for Random Forest

Figure S3. The Confusion Matrix for Random Forest

Figure S4. Radial Decision Tree Visualization: nodes represent decision points, colored by impurity (darker blue indicates higher impurity). Edge thickness highlights gain importance (thicker lines for higher gain), and node size corresponds to decision depth. The first and second percentages in each bracket show the portion of patients without and with events, respectively.
